# What determines inclusion in the early phase of the type 2 diabetes care trajectory in Belgium?

**DOI:** 10.1186/2049-3258-72-29

**Published:** 2014-08-25

**Authors:** Katrien Vanthomme, Nathalie Bossuyt, Sarah Moreels, Nicole Boffin, Etienne De Clercq, Geert Goderis, Viviane Van Casteren

**Affiliations:** 1 Scientific Institute of Public Health, Health Services Research Unit, Brussels, Belgium; 2 Université Catholique de Louvain, Institut de Recherche Santé et Société (IRSS), Brussels, Belgium; 3 Katholieke Universiteit Leuven, Leuven, Belgium

**Keywords:** Type 2 diabetes mellitus, Health services research, Chronic care, Family practice, Sentinel surveillance

## Abstract

**Background:**

In 2009, the Belgian National Institute of Health and Disability Insurance established a care trajectory (CT) for a subgroup of type 2 diabetes mellitus patients (T2DM) based on Wagner’s chronic care model. The goal of this CT is to optimise the quality of care using an integrated multidisciplinary approach. This study aims to identify patient-related factors associated with inclusion in a CT and to determine the most frequent reasons for non-inclusion.

**Methods:**

In 2010, the Belgian Sentinel Network of General Practices conducted a prevalence study of type 2 diabetes. The surveillance study carried out by this nationwide, representative network collected unique information about eligibility for the CT, inclusion in the CT and reasons for non-inclusion.

Based on the official inclusion and exclusion criteria, we first identified a group of eligible patients. Within this group, we then calculated the proportion of patients included in a CT as well as the prevalence of reasons for non-inclusion as reported by GPs. Furthermore, bivariate associations between patient-level parameters and inclusion were analysed. Finally, any patient-level parameters found to be statistically significant were included in a multivariate logistic regression model.

**Results:**

The 2010 study recorded 4600 Belgian type 2 diabetes patients. According to the official criteria, 589 patients were eligible for inclusion in a CT T2DM. By the end of August 2011, 95 patients had been included in a CT T2DM.

Our findings reveal that the younger the eligible patient was, the more likely he or she was to be included in a CT. Patients living in Flanders were more likely to be included in the CT than were patients living in Wallonia. Motivated patients with specific plans to change their diets were also more likely to be included in a CT.

The two most frequently reported reasons for non-inclusion were participation in another diabetes care programme and the timing of this surveillance study (inclusion will take place in the near future).

**Conclusions:**

Eligible diabetes patients who were admitted to a CT T2DM during the early phases of CT implementation were mainly found to be those who are able to make progress in their disease trajectories. In the future, more attention could be paid to also include more high-risk patients.

## Background

As a result of higher life expectancies, the prevalence of chronic diseases is increasing. The management of chronic diseases poses an enormous challenge to our health care system [1]. More than a decade ago, Wagner developed a chronic care model, which aimed to ameliorate the quality of chronic illness management within primary care. According to this model, the improvement of six interrelated components (i.e. self-management support, clinical information systems, delivery system redesign, decision support, health care organisation and community resources) should lead to interaction between informed, activated patients and prepared, proactive practice teams [2].

In 2009, the National Institute of Health and Disability Insurance (NIHDI) in Belgium introduced care trajectories (CT) for type 2 diabetes mellitus (T2DM) and chronic kidney disease based on this chronic care model. The aims of these CTs are to organise, coordinate and plan patient monitoring; to stimulate interaction and collaboration between patients, GPs, specialists and other caregivers; and to optimise the quality of care [3]. During the first CT phase, collaboration among patients, GPs and specialists is formalised in a four-year contract. The benefits of a CT for patients include receiving high quality care by means of a personal care plan; having access to information, materials and consultations; and receiving financial benefits. GPs play a coordinating role in the CT, supported by local multidisciplinary networks (LMNs) set up for this purpose.

This paper addresses the CT T2DM. Subgroups of T2DM patients are eligible for a CT when they meet specific inclusion criteria, namely when they are on one or two insulin injections a day or when they are insufficiently regulated with a maximum dose of oral antidiabetics. At the start of the CT, the following patients are excluded: pregnant women and women hoping to become pregnant soon; patients with type 1 diabetes; patients incapable of visiting the GP’s practice; and patients living in a rest home [3].

This study took place during the early phases of CT T2DM implementation. The goals of the study were to estimate the proportion of Belgian T2DM patients eligible for the CT, the number of eligible patients who had already been included at this early stage of CT implementation and the reasons behind non-inclusion. Gathering and analysing such information is crucial to the evaluation of this new approach to chronic care management, not only in Belgium but also internationally since the exchange of expertise in this new domain is essential [4]. Especially the study of the accessibility is important since every chronic patient should have an equal chance to be monitored well.

In particular, the research questions assessed in this paper are:

1. What proportion of T2DM patients are eligible for a CT based on the official inclusion and exclusion criteria?

2. What proportion of eligible patients had already been included in a CT T2DM by the end of this survey?

3. If a patient was eligible but had not been included, what reasons were reported by the GP for this non-inclusion?

4. Which patient-related factors are associated with inclusion in the CT T2DM?

## Data and methods

### Data collection

In 2010, a prevalence study of type 2 diabetes was conducted by the Belgian Sentinel Network of General Practices (SGP). This nationwide surveillance system was developed more than 30 years ago in line with similar sentinel networks in Europe that have a low turnover rate and voluntary participation. It has proven to be a reliable surveillance system for a wide range of health-related data, including diabetes [5-7]. The sentinel network is distributed well across the various districts of Belgium and was found to cover 1.5% (165,008 patients) of the Belgian population in 2010. The age and sex distribution of the sentinel GPs is also representative of the total Belgian GP population [8].

The SGP study included all patients diagnosed with type 2 diabetes before 2010. During the first phase of the study, GPs were asked to group together their T2DM patients, recording only the first contact with each patient in 2010 in order to avoid patients being recorded twice. In this way, the prevalence of type 2 diabetes could be estimated. Patients aged under 40 years were considered to be type 1 diabetes patients, and were therefore excluded. During this first phase, the following parameters were recorded for all T2DM patients: a patient identifier attributed by the GP, age, sex, year of diagnosis, treatment (only diet, oral antidiabetics or insulin), smoking status and physical activity.

The second phase of the study was implemented one month after the baseline data collection. In this phase, the GPs were asked to fill in any missing parameters from the first phase and to provide additional information about their perceptions of the patients’ motivation to improve their diet and stop smoking.

The third phase of the study took place in autumn 2011. Based on the information available on the patients’ treatment (i.e. the official inclusion criteria for the CT), the group of T2DM patients eligible for inclusion in a CT was defined. The GPs were asked to provide supplementary data on the eligible patients related to the following parameters: current treatment (extended to include treatment combinations and the category ‘incretin mimetics’), consultation at the GP’s practice, inclusion in a CT, inclusion date, reasons for non-inclusion (sometimes with the option to add additional reasons), up-to-date LDL cholesterol and height measurements, and the three most recent weight, blood pressure and HbA1c values.

## Methods

The total type 2 diabetes population was our starting point in answering the first research question on the proportion of diabetics eligible for a CT. Based on the treatment data gathered during the first phase of the surveillance study, we identified a group of patients who met the official inclusion and exclusion criteria (see Background section, paragraph 3) and were therefore eligible for the CT T2DM. We then used this group of eligible patients to calculate the proportion already included in a CT at the end of the surveillance survey in order to answer the second research question.

To answer the third research question on the reasons given by the GP behind the non-inclusion, eligible patients not yet included in a CT T2DM were identified. The GPs’ reasons for non-inclusion were categorised and measured for prevalence, and 95% confidence intervals (95% CI) were then calculated. GPs had been allowed to provide several reasons per patient, so the categories were not treated as mutually exclusive.

Finally, to answer the fourth research question on patient-related factors associated with inclusion in the CT, we examined the association of each relevant patient-level parameter (age, sex, region, duration since diagnosis, smoking status, physical activity, motivation to change diet and the most recent values attained for the clinical parameters) to the dichotomous outcome parameter (inclusion in a CT). Bivariate analysis was performed by means of chi square tests (or Fisher exact if N of the lowest cell <10) for the categorical variables and t-tests for the continuous variables. Furthermore, the percentage of patients included in a CT (+95% CI) was calculated for each category of the patient-level parameters. Finally, all variables which were found to have a significant (p < 0.05) bivariate association with the outcome were all at once included in a logistic multivariate model. Age and sex were included in the model by default. We also tested a multilevel model that included the patient-related variables nested in two levels, being the GP as one level nested within a local multidisciplinary network as an additional higher level. However, this multilevel did not produce a better data fit than the multivariate model and was therefore withdrawn.

## Results

### What proportion of T2DM patients were eligible for a CT based on the official inclusion and exclusion criteria?

In 2010, 4600 Belgian type 2 diabetes patients were recorded. Based on the inclusion criteria described above, 674 patients were eligible for inclusion in a CT T2DM. Of these 674 patients, 85 patients were excluded on the basis of the following exclusion criteria: desire to become pregnant (n = 1); living in a rest home (n = 15); incapable of visiting the GP’s practice (n = 65); and the combination of living in a rest home and being incapable of visiting the GP (n = 4). The final number of patients eligible for a CT T2DM was 589 (13% [95% CI: 12-14]).

### What proportion of eligible patients had already been included in a CT T2DM?

By the end of the surveillance study in August 2011, 95 of all eligible patients had been included in a CT T2DM (16% [95% CI: 13-19]).

### What reasons were reported by GPs for the non-inclusion of eligible patients?

Table 1 shows the main reasons given by GPs for the non-inclusion of eligible patients in a CT. In more than half of all cases, the patient was not included in the CT because he or she had already been admitted to an existing diabetes care programme (diabetes convention or the restricted programme for glycaemic control). According to GPs, 17% of the non-included eligible patients were to be admitted to a CT in the near future. In 15% of cases, GPs reported that inclusion was not desirable and/or that the patient was not motivated enough to be included in a CT.

**Table 1:** Reasons given by the Sentinel GPs for non-inclusion of eligible patients in the early phase of the Belgian diabetes type 2 care trajectory (August 2011)

	**N**	**% (95% CI)**
Patient is included in diabetes convention	179	49 (44-54)
Patient will be included in the near future	63	17 (14-21)
Inclusion not necessary according to the GP	28	8 (5-11)
Patient is not compliant, motivated	27	7 (5-11)
Other reasons	18	5 (3-8)
Patient will not sign the contract	17	5 (3-7)
GP will not sign the contract	17	5 (3-7)
Patient is included in the restricted programme for glycaemic control	13	4 (2-6)
Patient does not want to consult a specialist	7	2 (1-4)
Too much administrative work	6	2 (1-4)
Patient lives (partially) abroad	5	1 (1-3)
Specialist will not sign the contract	4	1 (0-3)

Information for 366 of the 494 eligible patients not included in a CT.

Categories are not mutually exclusive.

### What patient-related factors are associated with inclusion in the CT T2DM?

Table 2 shows the results of the bivariate and multivariate analyses. In Flanders, one in four eligible patients were included in a CT T2DM. This was a significantly higher percentage than in Wallonia, where only 6% of eligible patients were included. Motivated patients with specific plans to improve their diet were more often included in a CT (23%) than less motivated patients. Almost one in three patients whose most recent HbA1c values were well balanced were included in a CT. This was a significantly higher percentage of inclusion compared to patients whose HbA1c results were 7% or over.

**Table 2:** Patient-related factors associated with inclusion in the early phase of the Belgian diabetes type 2 care trajectory

	**Bivariate**			Multivariate*	
	**Mean**	**(95% CI)**	**p-value**	**OR**	**p-value**
Age	68	(65-70)	ns	0.96	0.016
Diabetes duration in years	12	(10-13)	ns	-	-
	**%**	**(95% CI)**	**p-value**		
(Men)	15	(11-19)			
Women	17	(14-22)	ns	1.62	ns
(Flanders)	24	(19-28)			
Wallonia	6	(3-9)	0.000	0.17	0.000
Brussels	15	(7-28)		0.47	ns
(Never smoked)	16	(12-20)			
Quit smoking	17	(13-23)	ns	-	-
Smoker	16	(9-26)		-	-
(Insufficient physical activity)	14	(11-19)			
Sufficient physical activity	18	(14-22)	ns	-	-
(Specific plans to change diet)	23	(17-29)			
Motivated to change diet but no plans yet	12	(7-21)	0.035	0.35	0.007
No plans to change diet	14	(9-20)		0.55	ns
(Most recent BMI < 25 kg/m^2^)	14	(7-26)			
Most recent BMI ≥ 25 kg/m^2^	27	(22-32)	ns	-	-
(Most recent blood pressure ≤ 130/80 mmHg)	23	(18-30)			
Most recent blood pressure > 130/80 mmHg	22	(17-28)	ns	-	-
(Most recent HbA1c < 7.0%)	30	(22-39)			
Most recent HbA1c ≥ 7.0%	20	(15-25)	0.022	0.50	0.027
(Most recent LDL cholesterol ≥ 100 mg/dl)	23	(17-30)			
Most recent LDL cholesterol < 100 mg/dl	23	(18-29)	ns	-	-

(Reference category).

ns = not significant.

CI: Confidence Intervals.

*N = eligible population. Due to missing values: age (n = 4), diet (n = 136), HbA1c (n = 178): N multivariate model: 327.

-: not included in multivariate model.

In the multivariate model, all of the above-mentioned associations remained statistically significant. Patients living in Wallonia were less likely to be included in a CT than patients living in Flanders; patients who had made specific plans to improve their diet were more likely to be included in a CT than patients who were motivated to improve their diet but had not yet made specific plans; patients with elevated HbA1c values were less likely to be included in a CT. This model also revealed that the older the patient was, the lower their odds of being included in a CT T2DM.

## Discussion

At the end of this surveillance study, in August 2011, we found that 16% of all eligible patients had been included in a CT. This percentage initially appears low, but should be seen in perspective. One important issue is the timing of this study. As the CT T2DM started on 1 September 2009, the 2010 study could reflect only the situation in the early phases of CT implementation. The timing issue was also reflected in the non-inclusion reasons given by GPs, where it was noticed that 17% of non-included eligible cases would be included in the near future. Furthermore, the official NIHDI figures, show that a considerable number of diabetes patients had already been included in a CT T2DM during this early phase of CT implementation [4].

Additionally, more than half of the non-included eligible patients had already been admitted to a different diabetes care programme. We can assume that if patients are managing well on an existing diabetes care programme, they will be less motivated to switch to the CT. Nevertheless, there are major differences between the two programmes; in that GPs play a far more central role in the CT and also that the CT takes a more multidisciplinary, patient-centred approach. Data from the Intermutualistic Agency which consists of administrative and reimbursement data of all sickness funds in Belgium, show that 28% of CT patients had made the transition from the diabetes convention to the CT [9]. In addition, the CT has already covered more patients than the long-standing diabetes convention category 3A.

Another important remark is that the CT’s exclusion criteria have changed since our study was conducted. Patients who are incapable of visiting a GP and those living in rest homes are no longer denied access to a CT. This makes the CT more accessible to patients who are less mobile and perhaps already in the later stages of disease. The adaptation of the exclusion criteria and the early timing of the study are two issues that may make it desirable to repeat the surveillance study at a later stage.

Since the CT T2DM was introduced to improve the quality of care for diabetes patients [2], it is important that all eligible type 2 diabetes patients have equal chances of being included. Yet, due to the study setting, we did not have any information on social determinants. It would be however interesting to study the association between social determinants and inclusion as is it likely that the socially most vulnerable patients are excluded the most. However, in our study we observed that younger patients, patients living in Flanders (vs. patients living in Wallonia), patients motivated to change their diet and patients with better HbA1c values were more likely to be included in a CT. The positive influence of a younger age and better motivation could be explained by the fact that GPs are more likely to include patients who are certain to make progress in their disease trajectory. It is inherent to the CT approach that patients need to make an effort to obtain a healthier lifestyle by means of their personal care plans. Lack of patient motivation was also one of the reasons given by GPs for non-inclusion. Results from the German disease management programme for diabetes also reveal that patients who have a lower risk of diabetes complications, higher self-activity rates and higher GP-rated motivation were more likely to be included in the programme [10]. This implies that in later stages of the CT, more attention could also be paid to less ‘obvious’ groups for inclusion.

However, the regional CT inclusion discrepancy is more closely related to structural issues. In our data, we observed that Flemish GPs are more likely to include their patients in a CT, whereas Walloon GPs are more likely to enrol their diabetes patients on a different diabetes care programme (i.e. the diabetes convention). These differences can also be observed in the real numbers of the NIHDI [9], which proves that our data are reliable. It is generally observed that differences exist between the approaches adopted by Flemish and Walloon GPs, and this is a fact that is also reflected in our annual GP surveys [8].

The key strength of this SGP surveillance study on the prevalence of diabetes lies in the fact that we were able to determine the percentage of patients eligible for a CT T2DM, the percentage already included in a CT T2DM and the reasons given by the GP for non-inclusion. This is unique information that is not available in electronic health records or elsewhere. It has been shown previously that the SGP is representative of Belgian GPs as a whole, which means that this study provides valid data about the early phase of CT implementation [11]. As mentioned above, it would be advisable to monitor these data on a regular basis in order to track any changes over time, which is crucial information for policy-makers. This kind of research is also indispensable for other countries implementing the chronic care model in their health care management systems, as little research has been conducted to date on such care programmes’ accessibility. A lesson for the future and for other countries as well could be to pay more attention to the inclusion of high-risk patients that are already in a later stage of their disease.

## Conclusions

Eligible diabetes patients who had already been included in a CT T2DM in the early phases of CT implementation were mainly those thought to be able to make progress in their disease trajectory. Younger patients and more motivated patients, in particular, were more likely to be included in a CT. Substantial regional differences were observed, with Flemish GPs more likely to enrol their diabetes patients on a CT.

The two most common reasons given by GPs for non-inclusion were participation in another diabetes care programme and the indication that inclusion would occur in the near future.

## Competing interests

The authors declare that they have no competing interests.

## Authors’ contributions

KV conceived the study, analysed and interpreted the data and drafted the manuscript. NB was involved in the study conception, the interpretation of the data and the manuscript revision. SM was involved in the study conception, the interpretation of the data and the manuscript revision. NB participated in the revision of the manuscript. EDC was involved in the study conception and design, the interpretation of the data and the revision of the manuscript. GG participated in the revision of the manuscript. VVC coordinated the study conception, study design and data collection. All authors read and approved the manuscript.
